# Supplementary material for: Are carbon emissions trading and green financial instruments synergistic? -Comprehensive quantitative research based on content analysis
Source: PLoS One. 2024 Mar 7;19(3):e0298601. doi: 10.1371/journal.pone.0298601 (PMC10919601; doi:10.1371/journal.pone.0298601)
Supplement: S1 Data — (ZIP) [file pone.0298601.s001.zip › Data/地级市Z_F工作报告.zip!/┴╔─■/╠·┴δ╩╨/└┤╫╘╣1⁄2╓┌║┼í╛┬φ┐╦╩2╛▌═°í┐_2015.docx]

各位代表：

现在，我代表市人民政府向大会报告工作，请予审议，并请市政协委员和列席同志提出意见。

一、2014年工作回顾

刚刚过去的2014年，是各种矛盾交织、面临困难最多的一年，也是顶住巨大压力、负重奋力前行的一年。我们在省委、省政府和市委的正确领导下，团结和带领全市各族人民，统筹做好稳增长、调结构、促改革、惠民生、防风险各项工作，努力保持全市经济社会平稳发展。

——国民经济基础进一步夯实。

预计，全市地区生产总值实现1080亿元，增长2%；

公共财政预算收入93.4亿元，为调整后预算的104.8%；

固定资产投资完成750亿元，下降28.2%；

规模以上工业增加值实现300亿元，增长2%；

社会消费品零售总额实现392亿元，增长12%；

城镇居民人均可支配收入19365元，增长10%；

农村居民人均可支配收入10985元，增长10.5%。

主要经济指标与铁岭实际状况进一步接近，经济发展质量进一步提升。

——开放步伐逐步加快。今年是项目开复工最早、检查次数最多、工作措施最实的一年，茅台保健酒、超高压水射流、石油机械三期等291个亿元以上项目开工建设，金飞马制漆、莱尼线束、比科电池等116个项目竣工。铁岭保税物流中心建设首次写入国务院文件，已获国家四部委批复并启动实施。调兵山市晓明镇独立工矿区、银州区铁西老工业区2个国家级搬迁改造试点积极推进，15个项目获国家支持，到位资金2.3亿元。招商引资成效显著，中石化地热开发、中电投热电联产、捷安特自行车等一批世界500强和全球知名企业投资项目落户铁岭。累计到位内资600亿元，实际利用外资5.5亿美元，向上争取各类无偿资金122亿元。外贸出口呈现新亮点，东电一公司成为首家出口创汇突破1亿美元的企业，辽宁石油机械签订三年6亿美元的对俄供货合同。全市出口总额实现7.2亿美元，增长12.5%。

——各项改革全面推进。取消和下放行政职权447项，行政审批及收费事项基本进驻公共行政服务中心，农村便民服务室在全省率先实现全覆盖。对2346家事业单位进行了分类确认，五大系统及相关工作职能顺利交接。市级食品药品监管机构重新组建完毕。工商登记制度改革全面推进，新登记各类内资市场主体18731户。在全省率先尝试混合型经济发展模式，国宏物业公司以联合入股方式成功收购铁岭金融护卫中心。我市被确定为国家基层卫生综合改革重点联系城市，基本药物配送率和入库率均达90%以上。新组建医疗纠纷调解委员会，建立了医疗纠纷第三方调解新模式。公车改革准备工作基本完成。各县（市）区实现本级国库支付全覆盖。农村土地承包经营权确权试点工作有序推进。首批5个扩权强镇试点开始启动。

——工业经济运行质量稳步提升。出台11项惠工政策，推进兜底服务常态化，双百企业提质增效步伐加快。九三粮油、曙光集团销售收入双双突破50亿元，税收超千万元企业达到20户，地方工业增值税增长16%，全市工业用电量增幅跃居全省第3位。泰石岩棉、煤机装备制造、万隆包装等一批规模实力强、装备水平高、产品技术领先的重大工业项目开工建设。6户企业在上海股权交易中心挂牌，24户企业在科技板挂牌。专用车、换热设备等8个省级产业集群销售收入增长8%，煤机装备产业集群获批为国家火炬特色产业基地。科技创新迈出新步伐，申请专利700件以上，实施产学研合作项目50项，圣加仑等10户企业被评为国家级高新技术企业，北宇真空获得全省首台套项目奖励，58户企业进入省创新型中小企业行列。

——城乡建设水平进一步提高。新三线公路二期开工建设，维修改造县级以上公路479公里、农村道路820公里。城市总体规划修编工作扎实推进，规划土地管理职能进一步规范。凡河城区功能日臻完善，千山路商业街、长白山路农贸市场、儿童乐园、公共自行车等项目投入运营，单户城镇化期房主体完工，光荣街南段道路工程启动建设，商业企业和个体工商户发展到1550户。银州区城市管理体制进一步理顺，岭东热源厂二期投入运营，长青路顺利贯通，26条主次干道改造、中茂人防商业大道地上工程完工。开原滨水新城整体框架已经拉开，昌图滨湖新城部分房地产项目实现入住，辽北新城起步区初见形象，西丰县环境卫生整治成效显著，调兵山市城市品位进一步提升，清河区被确定为省级国家养老产业示范城市。新台子、八面城、庆云堡等中心镇建设取得新进展。

——农业农村经济形势喜人。粮食总产量达到92.8亿斤，创历史新高。一县一业特色产业规模不断壮大，我市获全国林业产业突出贡献奖。高标准养殖基地发展到40个，32个标准化畜牧场通过国家示范创建验收，畜牧业产值实现290亿元，占全省的15%。辉山乳业、益海嘉里二期等投资10亿元以上大项目建设进展顺利，农业产业化总产值实现762亿元，农民人均从产业化链条上获得收入7000元。新型农业经营主体快速发展，农民合作社数量居全省第一位。土地流转面积达到260万亩，占家庭承包地的38%。新增节水滴灌面积15.5万亩，农业全程机械化水平提高到80%，农村气象灾害防御体系基本建成。建立科技示范基地30个，农业标准化生产面积达到580万亩，新增羽叶、鹿源2件中国驰名商标，国家地理标志保护产品达到12个。支持西丰发展实现重大突破。

——服务业四年行动计划开局良好。莲花湿地景区晋升省级服务业集聚区。东北参茸中草药材市场被评为商务部流通追溯体系建设试点单位。星悦南岸商住小区、全球名品购物中心、新浪潮水世界等项目主体完工。东北城农资物流园、辽宁二手车交易市场、时代广场、禧徕乐家居、东北亚花木城等项目投入运营。旅游业“一区四带”发展格局初步形成，天力酒店、凡河生态谷、龙源温泉度假村、城子山风景区等一批特色项目开发建设，全国荷花展暨荷花文化旅游节系列活动成功举办，吸引周边城市百万游客游新城。金融广场投入运营，信达财险、阳光财险入驻铁岭，新发展小贷公司9户，财京公司、开原市企业债成功发行，金融机构各项存款余额首次突破1000亿元，金融业营业税增长46%。新开发房地产面积1470万平方米。实施工业企业主辅分离81户，新立生产性服务业企业84户。成立全省首家市级电子商务协会，78家电商企业入会。新建农户科学储粮仓4000座，全市粮食市场保持稳定。

——生态文明建设成效显著。国家节能减排财政政策综合示范工作扎实推进，5个方面典型示范项目获国家批复，到位中央奖励资金4亿元，节能和主要污染物减排任务顺利完成。亚泰水泥3条生产线完成脱硝改造，铁煤热电厂1号机组脱硫改造工程主体完工。启动实施凡河水系保护三年计划，辽河保护区建设取得重大进展，辽河铁岭段水质稳定达到Ⅳ类标准。完成植树造林11万亩，治理闭坑矿山956亩。农村畜禽粪污治理工程全面启动，7个有机肥生产示范企业投产。建设市级宜居示范村47个，创建省宜居示范乡镇13个。我市被确定为全国水生态文明建设试点市和首批省级环保模范城市。

——社会事业繁荣发展。成功举办首届东北二人转邀请赛、高鹗和《红楼梦》学术研讨会、纳兰性德奖和端木蕻良奖文学创作大赛等文化活动，铁岭欢歌广场文化品牌进一步叫响。朝鲜族秋夕节列入国家非物质文化遗产保护名录。清华大学工笔画高研班在我市办学。完成义务教育学校标准化建设88所，新建、改扩建标准化幼儿园10所，现有校车全部安装卫星定位系统。我市被评为中国教育优先发展示范城市，连续三届获得全国教育改革创新特别奖。调兵山市、开发区进入全国义务教育发展基本均衡县区行列，清河区通过省政府基础教育强区评估验收，西丰县义务教育营养餐工作经验在全省推广。新农合大病保险全面启动，公共卫生服务水平进一步提升。新家庭计划省级试点工作开始实施。成功举办自然水域垂钓、围棋甲级联赛、体育舞蹈、软式棒垒球等国家级体育赛事。我市培养输送的运动员夺得3项世界冠军、1项亚运会冠军并打破1项世界纪录。市档案馆、市残疾人康复中心投入使用。学习型城市创建工作深入推进，国防动员和双拥共建工作得到加强，精神文明建设取得新成效。

——社会形势保持稳定。年初确定的十件民生实事扎实推进。实施保障性安居工程18166户、暖房子工程405万平方米，新建和改造街路111条、垃圾场站102个，中心城区集中供热率提高到97%。为850户残疾人家庭进行了无障碍改造，为937个行政村安装太阳能路灯1.4万盏。靠山屯棚户区居民喜迁新居，沙子沟棚改回迁楼、莲花湿地三期主体完工，铁岭大剧院、美术馆启动建设。安置实名制就业59917人，城镇登记失业率控制在3.2%以内。城镇五险参保人数达到202.2万人次，新农保与城镇居民基本养老保险并轨，各项保险和供养标准全面提高。集中治理弃管小区52个、弃管楼582栋。70个农村互助幸福院、15个社区老年人日间照料中心投入使用。解决了11.4万农村人口饮水安全问题，6.5万农村贫困人口脱贫。市县乡三级干部全员接访工作机制、社会矛盾纠纷大调解体系基本建立。应急管理和安全生产责任体系、监管能力、制度建设全面加强。成立全省唯一平安文化教育基地，立体化治安防控体系进一步健全，群众安全感连续三年保持全省第一。

民族宗教工作取得新进展，清河区政府被评为全国民族团结进步先进集体。12345市长服务热线运行良好，政务公开成为新常态，政务督查和绩效管理工作得到加强，依法行政水平明显提升，政府廉政建设取得新成效。第二批党的群众路线教育实践活动高质量完成，为加强政府自身建设提供了保证。全年办理人大议案、人大代表建议和政协提案496件，见面率、办复率和满意率均达100%。

外事、侨务、统计、审计、物价、综合执法、国税、地税、商检、烟草、国家安全、武警、消防、地震、邮政、通信、驻外联络、市志、老龄、仲裁等工作也都取得了新成绩。

过去一年的成绩来之不易，这是省委、省政府和市委正确领导的结果，是市人大依法监督、市政协民主监督和大力支持的结果，更是全市广大干部群众智慧和汗水的结晶。在此，我代表市人民政府，向辛勤工作在各条战线上的全市各族人民，向积极支持政府工作的市人大代表、市政协委员、各民主党派、各人民团体、中省直驻铁单位和社会各界人士，向解放军驻铁部队指战员和武警官兵，向所有关心、支持、参与幸福美好新铁岭建设的海内外朋友，表示衷心的感谢并致以崇高的敬意！

在看到成绩的同时，我们也清醒地看到经济社会发展中存在的问题和挑战，特别是地区生产总值、公共财政预算收入、 固定资产投资等指标未能完成年初计划，有的指标出现了明显下滑。这既有宏观经济形势深刻变化、市场需求疲软带来的下行压力，也有我市产业结构不尽合理、体制机制不够完善形成的制约；既有落实宏观调控政策、主动适应政策性减收因素带来的影响，也有我们坚持数字做实、主动贴近铁岭实际出现的降幅。深入分析面临的矛盾和问题，主要表现在：一是经济增长缺少大项目支撑，一些县（市）区仍处在项目少、主导产业牵动力不强的状态，发展后劲不足。二是财政收入规模小，收支矛盾突出，人均财力和人均存款在全省排在靠后位置。三是政府债务特别是本级债务负担沉重，还本付息资金来源严重缺乏，偿债压力巨大。四是政府职能转变还不到位，市、区之间责权利不清，简政放权工作任重道远。五是政府自身建设还需加强，个别部门和少数人不愿为、不敢为、不能为的现象程度不同的存在，软环境建设仍有不尽人意之处。这些问题已引起我们高度重视，并将在今后工作中认真加以解决。

二、2015年的主要工作

今年是实施“十二五”规划的收官之年，也是全面深化改革、推进依法治市的关键之年，改革发展稳定任务之重前所未有，矛盾风险挑战之多前所未有，改善民生投入压力之大也前所未有。政府工作的指导思想是：深入贯彻落实党的十八大及三中、四中全会和中央经济工作会议精神，主动适应经济发展新常态，坚持稳中求进总基调，牢固树立实事求是、依法行政理念，推进新型工业化、信息化、城镇化、农业现代化同步发展，更加注重经济发展的质量和效益，更加注重转方式调结构，推进改革创新，强化风险防控，加强民生保障，奋力夺取幸福美好新铁岭建设的新胜利。

主要预期目标是：地区生产总值增长6%，公共财政预算收入增长2%，固定资产投资增长6%，社会消费品零售总额增长11%，城乡居民人均可支配收入分别增长10%以上。重点抓好以下10项工作：

（一）牢固树立项目立市理念，全面扩大对外开放

铁岭是投资拉动型的内陆城市，必须以原始资本积累为手段，靠项目积累完成总量的扩大。深入开展项目年活动，进一步落实项目工作责任制，坚持理性务实科学招商，努力在引进大项目、培育大企业、催生大产业上实现新突破，形成新的经济增长点和财税增收点。依托铁岭的区位、产业和资源优势，采取产业链招商、以商招商、飞地招商、目录招商等有效方式，主动承接沈阳、长春等东北城市和发达地区产业转移，努力引进投资规模大、科技含量高、产业一体化、能够支撑铁岭这片天地的大项目。全年到位内资650亿元，实际利用外资1.4亿美元（新口径）。抢抓国家新一轮振兴东北政策机遇，加快银州区铁西、调兵山市晓明镇搬迁改造试点项目建设，争取中石化地热开发、昌图古榆煤层气、红星美凯龙等央企民企沪企辽宁行项目开工建设，确保铁岭保税物流中心封关运行。全力推进煤机装备制造、泰石岩棉、捷安特自行车等一批在建项目，尽早实现投产达效。树立安商养商与招商并重的理念，切实加强软环境建设，集中政策资源扶持原有小项目做大、大项目做强。加快农畜产品、专用车和汽车零部件三大出口基地建设，努力打造国家级服务外包示范城市，进出口总额实现6.5亿美元（新口径）。

（二）全面深化各项改革，激发经济社会发展活力

围绕解决发展面临的突出问题推进改革，向改革要红利、要活力、要动力。进一步健全政府派出机构的管理体制和运行机制，赋予各县（市）区相应的事权、财权、行政权，形成责权利相统一的行政运行模式。加大简政放权力度，积极稳妥推进县级政府机构、事业单位、园区管理体制和公车改革，理顺工商、质监划归地方后的体制机制问题，加快建立负面清单和行政审批网络平台，提高行政效能和服务水平。切实转变政府职能，创造更好的市场竞争环境，培育市场化创新机制，营造有利于大众创业、市场主体创新的政策环境和制度环境，在维护公平、改善金融支持、强化激励机制、集聚优秀人才等方面积极作为。实施市县食品药品监管、卫生计生体制改革，推动卫生与计生资源的有效融合。加快推进国资国企改革，抓好铁岭精工、铁阀集团搬迁改造和北方铜业转型转产。完成集体林权主体改革任务，搞好土地承包经营权确权登记颁证工作。抓好农信社产权制度改革，筹建银州区和调兵山市农商行。继续做好5个扩权强镇试点的权力下放和承接工作。稳妥调整行政区划，合理布局城市发展空间。深入开展调研论证，科学编制“十三五”规划。

（三）大力实施工业强市战略，进一步做大工业经济

把做大工业作为今后相当长时期工作的重中之重，坚持引进大项目与做大现有企业并重、调整产业结构与促进产业升级并重，积极主动为中省直企业、合资合作企业和民营企业搞好服务，真正把企业的事当成政府的事来办，提振企业发展信心。深入实施双百工程，重点扶持九三粮油、曙光集团等销售收入前100户大企业，下力气培育圣加仑、哈深冷等有前景的100户中小企业。大力发展产业集群，抓好金飞马制漆、石油机械工业地产等项目建设，提升上下游产业配套能力，引进战略投资者对现有企业进行兼并重组，实施重点企业技改项目100个，推动传统产业向中高端升级。拓宽工业发展领域，重点支持乾丰电动大巴、比科电池、翰博太阳能电动车等新兴产业项目建设，推动中小微企业向生产小型化、智能化、专业化方向发展，促进信息化与工业化融合。继续搞好域内企业协作配套，加大政府采购地方工业产品扶持力度。把创新作为驱动发展新引擎，有效利用中科院等研究单位和高校科技资源，积极开展技术转移转化及相关工作，完成新产品新技术开发500项，新增省级技术中心3个，以产业化的创新培育新的增长点。

（四）坚持数量质量效益并重，加快转变农业发展方式

铁岭的最大优势是农业，必须靠农业的工业化（产业化）实现工农业互相促进，靠发展农业的休闲旅游实现一三产业互动，靠土地流转实现规模化经营和劳动力转移，逐步实现农业产业化、农民职业化、农村城镇化。继续实施百亿斤粮食产能工程，大力推广优良品种和先进实用技术，增强气象灾害防御能力，加快推进玉米、水稻全程机械化和节水滴灌工程建设，提高粮食和蔬菜、榛子、苗木等主要农产品的产量和质量，打造特色农业品牌。以建设全省生态畜牧业示范市为目标，新建和改造50个规模化标准生态养殖场，做大做强雨润、嬴德、中敖等一批有影响力的龙头企业，畜牧业产值力争达到320亿元。大力发展农产品精深加工，重点抓好投资10亿元以上大项目，力争北大荒集团（昌图）项目签约落地，辉山乳业、茅台保健酒等项目建成投产，规模以上龙头企业销售收入达到720亿元，打造全国食品工业基地。注重农业科技创新，实施与省农科院的全面科技合作，加快建设辽宁铁岭国家农业科技园区，促进依农科技等企业发展壮大。抓好“三品”认证、绿色食品原料基地和农业标准化示范区建设，提高农产品质量安全水平。大力推广秸秆综合利用，使秸秆资源变废为宝。加快发展农民合作社、家庭农场等新型农村合作经济组织，推动土地规模经营，新增土地流转面积40万亩。组建市县农村综合产权交易中心，吸引工商资本向农业领域投入。继续举全市之力支持西丰发展，力争早日实现富民强县目标。

（五）强化服务业支撑作用，逐步提高三产对经济增长的拉动力

把提升质量和做大规模作为今后服务业发展锲而不舍的目标，依托大型载体落地实现产业扩张，依托一产转移、二产拉动实现产业扩张，依托新型产业发展、力推工商企业网上销售实现产业扩张，进一步释放消费潜力。加快发展现代物流产业，重点支持东北物流城大项目引进和店铺招商，打造特色鲜明的专业市场集聚区。做大7S汽车广场、二手车交易市场及配套产业规模，加快推进汽车嘉年华项目，打造辐射东北地区的汽车产业综合体。抓好星悦南岸项目建设，力争全球名品折扣店、新浪潮水世界投入运营，建成吸引高端消费的时尚中心。深入挖掘整合旅游资源，规划一批精品旅游线路，保护开发一批历史文化遗产遗迹，发掘一批民间艺人和民俗，开发一批生态旅游品牌项目，建设一批现代旅游景区景点。重点抓好莲花湿地景区、凡河生态谷、城子山风景区、龙源温泉度假村等龙头项目，搞好农家乐和乡村旅游，加强餐饮、住宿、购物等功能配套，推行网上预约预订服务，与大型旅行社开展合作，形成全天候、一条龙旅游服务体系，真正把游客吸引来、留得下、能消费，旅游总收入增长20%以上。加快发展粮食产业，力争在市场建设、精深加工和品牌培育上取得新成效。大力开发金融、养老、康复、商业等各类地产，新开发面积1500万平方米，新增保障性安居工程9446套。鼓励发展电子商务、美容健身、信息服务等新型产业，分离壮大生产性服务业，培育新的增长点。

（六）坚持量力而行原则，统筹推进城乡建设

坚持以人为本、遵循规律、因势利导、产城融合的理念，以组团式发展为方向，以提升城镇化质量为目标，积极稳妥推进与经济社会发展水平、资源环境承载能力相适应的新型城镇化，逐步缩小城乡生活水平差距。按照新区繁荣、老区提升、融合发展的思路，凡河城区重点完善城市功能，抓好天力酒店、恒基商业步行街等项目建设，加快完善交通设施和小区物业管理，扶持发展商贸服务业，进一步提升商气人气。银州区重点建设中茂人防商业大道、哥伦布广场等综合体项目，启动中国梦家园养老中心建设，加大街路改造、园林绿化、城市美化亮化力度，打造服务业核心区。积极推动沈铁二号线和沈铁城际铁路建设，高标准开发建设新台子新城，加快沈铁同城化步伐。进一步提高县城综合承载能力，重点实施昌图县辽北新城建设、开原市城市景观提升、西丰县寇河治理、调兵山市新开河综合治理、清河区大供热联网改造等工程，增强县城对农业转移人口的吸纳能力。加强中心镇建设，打造一批连接城乡、服务周边农村的关键节点。坚持建管并重，突出抓好城市精细化管理，努力建设宜居宜业、环境优美的城市。

（七）切实加强生态保护，努力建设美丽铁岭

顺应人民群众对良好生态环境的新期待，坚持治标治本多管齐下，朝着蓝天净水青山目标不断推进。充分利用国家节能减排政策，加快辽河生态文明示范区、江河湖泊综合治理、低碳城镇及典型示范项目建设。实施排污许可证制度，推进主要污染物排放初始权核定工作，加强重点行业节能改造和污染治理，确保完成“十二五”节能减排目标。抓好一县一热源建设，集中整治工业园区分散燃煤锅炉及重点领域扬尘污染，实施铁岭电厂、煤矸石电厂二期供热改造，淘汰3000辆黄标车和老旧车辆，努力提升空气环境质量。加快推进国家水生态文明建设试点工作，重点抓好凡河水系治理、污水处理设施运行、畜禽养殖污染治理及271个农业源减排项目，确保辽河水质持续改善。继续实施青山工程，不断提高造林绿化质量。积极推进宜居乡村建设，特别是加大环境卫生整治和村屯绿化力度，不断完善农村基础设施，建设美丽乡村。

（八）进一步做实财税收入，强化发展要素保障

积极应对国家财税政策调整，抓住税源建设不放松，加大向上争取项目、资金力度，最大限度地争取上级转移支付，提高财政综合保障能力。牢固树立过紧日子思想，强化开源节流、量入为出的理念，建立全口径预算体系和预算稳定调节基金，集中财力用于改善民生、促进发展等重点支出，严格控制一般性支出和预算追加，增强预算的硬约束力。按照严控增量、区别对待、分类施策、逐步化解的原则，进一步完善政府举债偿债工作机制，确保按期支付，维护政府信用。加大股份制银行引进力度，力争新设域外金融机构2家，小贷公司发展到45家，推动铁岭银行走出市门、发展壮大，形成金融机构为实体经济服务和竞争新格局，存贷款和非信贷融资分别增长10%以上。加强金融生态环境建设，完善政府融资平台基础要素，建立健全投融资责任体系，鼓励和引导社会资本向公共事业和基础设施建设领域投资。进一步抓好银企对接，积极推进企业直接融资，力争3-5户企业在股权交易中心挂牌，重点培育一批有希望上市的企业。加大不良企业和项目整治力度，妥善处置闲置低效用地，提高土地利用效率。做好单户城镇化期房入住、农村住房腾出和土地复垦工作，妥善处理金峰小镇等遗留问题。积极引进海外研发团队，深入开展校企对接，提高人力资源质量和技术水平，努力保障企业用工需求。

（九）更加注重改善民生，努力繁荣社会事业

按照守住底线、突出重点的要求，实施16件民生实事：1.全市维修改造农村道路790公里，新三线公路二期工程竣工通车。2.全市实施暖房子工程480万平方米，银州区暖房子改造工程基本完成。3.加强全市城区物业管理。4.全市维修改造弃管楼360栋。5.实施全市城区集中连片棚户区改造工程。6.提高全市新农合筹资标准，开展大病保险“一站式”服务。7.按省政府要求，提高全市城乡最低生活保障和农村五保户供养标准。8.实施全市困难家庭高校毕业生就业援助工程。9.为全市90周岁以上高龄老人提高生活补贴标准。10.为全市中小学安装视频监控系统，实现校园安全实时监控全覆盖。11.全市新建农村饮水安全工程80处。12.实施凡河城区黑龙江路连接102线道路建设工程。13.实施银州区广裕街亮化工程。14.新老城区连接线光荣街南段道路工程竣工通车。15.继续抓好铁岭大剧院、美术馆建设。16.实施银州区雨污分流改造工程。

大力扶持就业创业，进一步扩大社会保障覆盖面。继续开展教育年活动，推动县域义务教育均衡发展，加强师德和师资队伍建设，改善贫困地区办学条件。抓好学校D级危房改造，开展交通安全、法律知识进校园活动。加强城乡幼儿园标准化建设，让适龄儿童共享优质公平教育资源，不让孩子输在起跑线上。继续推动铁岭师专升本工作，统筹发展各级各类职业教育，坚持学校教育与职业培训并举，力争实现每个有劳动能力的铁岭人都有一技之长目标。加强城乡医疗卫生服务能力建设，努力提高全市人民健康水平。抓好贫困残疾人助行和家庭无障碍改造，提高社区基本康复服务水平。办好以历史名人命名的系列文化活动，实施一批文化惠民工程，建立一批文化产业基地。深入开展全民健身活动，办好铁岭市第八届运动会。鼓励和发展体育产业，促进体育消费。落实党的民族政策，依法加强宗教事务管理。加强国防动员和后备力量建设，全面落实双拥、优抚、安置等政策，实现全国双拥模范城“三连创”目标。建立安全生产信息化监管新模式和专家排查隐患新机制，做好食品药品监管工作，提高突发事件综合防范应对能力。深入实施平安铁岭四级联创，推动社区（村）网格化管理、社会化服务。提升公安信息化和警务实战化能力，夯实立体化治安防控体系。健全社会矛盾纠纷大调解工作机制，积极化解重点信访问题。推动孝道文化进家庭、进学校、进社区（村）、进机关、进企业，落实老年人体检、乘坐公交车、游览公园景点等优惠政策，逐步解决好老年人活动场所等实际问题，弘扬优秀传统文化。深入开展学习型城市和文明城市创建活动，在全社会培育和践行社会主义核心价值观，汇聚发展正能量。

（十）推进依法行政，建设法治廉洁务实责任政府

自觉接受市人大及其常委会的依法监督和市政协的民主监督，认真听取人大代表、政协委员、民主党派、工商联、无党派人士和各人民团体的意见，积极办理人大议案、人大代表建议和政协提案。强化宪法意识，大力推进依法行政，进一步规范执法行为，做到法定职责必须为、法无授权不可为，坚决克服懒政怠政，严肃追究失职渎职。健全依法决策机制，确保决策制度科学、程序正当、过程公开、责任明确。着力提高市长服务热线运行水平，强化政务督查和绩效管理，全面推进政务公开，让政府权力在阳光下运行。深入落实党风廉政建设一岗双责制度，增强各级干部的红线意识和底线思维。坚持以说实话、办实事、求实效为原则，做实政府的各项工作，抓实改革的各项重点，夯实发展的各项基础，落实民生的各项政策，使铁岭的经济社会得到量的扩张和质的提升，给子孙、给未来发展留资源、留环境、留空间。坚持一张蓝图绘到底，一个思路贯始终，不求政绩求实绩，不求己升求民生，始终保持时不我待的紧迫感、拼搏争先的责任感、不进则退的危机感，以昂扬向上的工作激情、务实争先的工作作风、奋发有为的精神状态，努力开创政府工作的新局面。

各位代表，铁岭的发展任重道远，人民的期待催人奋进。面对前所未有的机遇和挑战，勤劳睿智的铁岭人民一定有不甘人后的英雄气概，一定有在欠发达市情基础上建设经济强市的坚强决心，也一定有把铁岭推向全面开放的巨大能力。让我们更加紧密地团结在以习近平同志为总书记的党中央周围，在中共铁岭市委的正确领导下，凝神聚力，真抓实干，为早日实现幸福美好新铁岭宏伟目标而努力奋斗！
